# Trelagliptin stimulates osteoblastic differentiation by increasing runt-related transcription factor 2 (RUNX2): a therapeutic implication in osteoporosis

**DOI:** 10.1080/21655979.2021.1900633

**Published:** 2021-03-18

**Authors:** Haiyu Shao, Renzheng Wu, Li Cao, Haifeng Gu, Fang Chai

**Affiliations:** a Department of Orthopedics, Zhejiang Provincial People’s Hospital, People’s Hospital of Hangzhou Medical College, Hangzhou, Zhejiang, China; b Department of Orthopaedics, Dongyang Garden Tianshi Hospital, Dongyang, Zhejiang, China

**Keywords:** Osteoporosis, Trelagliptin, osteoblastic differentiation, RUNX2, AMPK, MC3T3-E1

## Abstract

Osteoporosis, an aging-associated bone metabolic disease, is affecting millions of people worldwide. The deregulated process of osteoblastic differentiation has been linked with the progression of osteoporosis. Trelagliptin is a long-acting inhibitor of DPP-4 used for the management of type 2 diabetes mellitus. However, it is unknown whether Trelagliptin possesses a beneficial effect in osteoblastic differentiation. Interestingly, we found that treatment with Trelagliptin enhanced differentiation and promoted the mineralization of MC3T3-E1 cells. Firstly, Trelagliptin increased the activity of alkaline phosphatase (ALP) and promoted osteoblastic calcium deposition. Additionally, treatment with Trelagliptin upregulated ALP, osteocalcin (OCN), osteopontin (OPN), and bone morphogenetic protein-2 (BMP-2). Notably, Trelagliptin increased RUNX2, a major regulator of osteoblastic differentiation. Mechanistically, Trelagliptin upregulated the levels of p-AMPKα. Blockage of AMPK with compound C abolished the effects of Trelagliptin in RUNX2 and osteoblastic differentiation, suggesting the involvement of AMPK. Our findings suggest that Trelagliptin might possess a potential for the treatment of osteoporosis.

## Graphical Abstract

**Figure:**
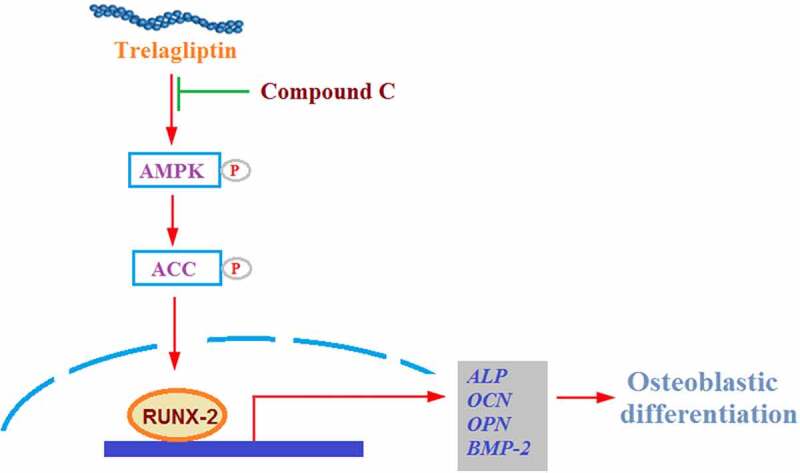

## Introduction

Bone loss is induced when the process of bone resorption exceeds bone formation, contributing to the pathogenesis of osteoporosis (OP) [1–3]. OP is defined as a systemic bone disease that results from decreased bone mass, increased osteopsathyrosis, declined bone mineral density (BMD), and elevated fracture risk [4]. Significant high morbidity of OP is reported in the elderly population and postmenopausal women. The global OP population is projected to reach 221 million before 2050 [5]. Currently, rare effective therapy is available for the treatment of clinical OP. The life quality of OP patients is significantly affected by such clinical characteristics as pains and tiredness that are induced by OP. Multiple factors are reported to be involved in the pathogenesis of OP, including genetic factors, transcriptional factors, signaling pathways, hormonal readiness, and cytokines [6,7]. Osteoblasts are the main components of the bone and are mainly differentiated from bone marrow mesenchymal stem cells (BMSCs), through a complicated process. Multiple signaling pathways are involved in the process of osteogenic differentiation [8], among which Runx2 is an important osteogenic transcription factor. Runx2 is reported to play a critical role in the early stage of osteogenic differentiation and has already been regarded as a differential biomarker [9,10]. Bone morphogenetic protein-2 (BMP-2) is reported to be closely related to bone formation [11]. It induces the osteogenic differentiation of BMSC by activating alkaline phosphatase (ALP) and elevating the expression level of osteocalcin (OCN) [12]. Dai reported that genistein induced osteogenic differentiation by regulating the BMP2/SMAD5/Runx2 signaling pathway [13], which is regarded as the classic BMP signaling pathway that regulates osteogenic differentiation through mediating the expression of Runx2. AMPK is an important factor that regulates osteogenic differentiation, although with controversy on its positive or negative feedback regulation mechanism on osteogenic differentiation [14–16]. Also, Runx2 proteasomal dependent-degradation is induced by the activation of AMPK [17]. Therefore, the AMPK-dependent signaling pathway is thought to be a potential target for the treatment of OP.

Trelagliptin is a long-acting selective inhibitor of DPP-4, developed by Takeda pharmaceuticals for the treatment of type 2 diabetes mellitus [18,19]. As an oral hypoglycemic drug, compared to insulin, treatment compliance of diabetic patients has been significantly promoted by Trelagliptin, which ameliorates the life quality of diabetic patients to a great extent [20]. Recently, DPP-4 has been reported to exert a regulatory effect on bone metabolism [21] and DPP-4 inhibitors have the ability to reduce osteoporotic fracture [22,23].

The potential mechanism of DPP-4 inhibitors could involve both promoting bone formation and reducing bone resorption through their substrates and related metabolism [21]. Diabetes patients are at high risk of developing the OP condition. We hypothesized that Trelagliptin could have a beneficial effect on diabetic-related OP. Here, the effect of Trelagliptin on osteoblastic differentiation, as well as the underlying mechanism, will be investigated to explore the potential therapeutic property of Trelagliptin on OP.

## Materials and methods

### Induction of osteoblastic differentiation

The MC3T3-E1 cells were obtained from ATCC (Virginia, USA) and were cultured in α-MEM medium (Gibco, New York, USA) at 37 °C. For the induction of osteoblastic differentiation, the cells were stimulated with osteogenic differentiation medium (OM) consisting of the α‐MEM medium, 4 mM β‐glycerophosphate, and 25 μg/mL ascorbic acid. To test the effect of Trelagliptin, MC3T3-E1 was incubated with OM media containing 50 μM Trelagliptin for 14 days.

### MTT assay

MTT assay was used to evaluate the viability of treated MC3T3-E1 cells. After treatment, cells were loaded with 5 mg/mL of MTT solution and incubated for 4 hours. Subsequently, the culture medium was removed and 200 μL dimethylsulfoxide (DMSO, Genview, Beijing, China) was added to dissolve the formazan. Lastly, the absorbance (OD value) at 590 nm was read.

### Real-time PCR analysis

MC3T3-E1 was incubated with OM media containing 50 μM Trelagliptin for 14 days. The TRIzol reagent (Invitrogen, California, USA) was used to extract total RNA from MC3T3-E1 cells and further transcribed into cDNA utilizing a Kit (Thermo, Massachusetts, USA) according to the instructions of the manufacturer. Subsequently, cDNA was applied for real-time PCR in a 20 μL PCR system containing a 10 μL 2× Power SYBR Green Master Mix (Invitrogen, California, USA). GAPDH was used to normalize the relative expressions of target genes and calculated using the 2-^ΔΔt^ method. The following primers were used: ALP (Forward: 5ʹ-GGGGACATGCAGTATGAATT-3ʹ, Reverse: 5ʹ- GGCCTGGTAGTTGTTGTGAG −3ʹ); OCN (Forward: 5ʹ-TGAACAGACTCCGGCG-3ʹ, Reverse: 5ʹ-GATACCATAGATGCGTTTG-3ʹ); OPN (Forward: 5ʹ-TGGAACTTGCTTGACTATCGA-3ʹ, Reverse: 5ʹ-GACCACATGGACGACGATG-3′);

BMP2 (Forward: 5ʹ-AACACCGTGCGCAGCTTCCATC-3ʹ, Reverse: 5ʹ- CGGAAGATCTGGAGTTCTGCAG-3ʹ); RUNX2 (Forward: 5ʹ- CTTCATTCGCCTCACAAAC-3ʹ, Reverse: 5ʹ-GTCACTGCGCTGAAGA-3ʹ),

GAPDH (Forward: 5ʹ-AGGTCGGTGTGAACGGATTTG-3ʹ, Reverse: 5ʹ- TGTAGACCATGTAGTTGAGGTCA-3ʹ).

### Western blot assay

MC3T3-E1 was incubated with OM media containing 50 μM Trelagliptin for 14 days. The cells were lysed using the cell lysis buffer (Thermo, Massachusetts, USA) to extract total proteins, and separated using the SDS-PAGE. Then, proteins were transferred onto the PVDF membrane, followed by being incubated with 5% BSA solution to remove the nonspecific binding proteins. Subsequently, the membrane was incubated with primary antibodies from the Cell Signaling Technologies (CST), USA, against Runx2 (1:1000), p-AMPKα (1:1000), AMPKα (1:1000), p-ACC (1:1000), ACC (1:1000), or β-actin (1:1000), followed by being incubated with horseradish peroxidase-conjugated secondary antibodies (1:1000, CST USA) for 1–2 hours. Lastly, the blots were incubated with ECL reagents (Thermo, Massachusetts, USA) and visualized using Image J software.

### ALP activity

MC3T3-E1 was incubated with OM media containing 50 μM Trelagliptin for 14 days, followed by conducting the ALP staining assay according to the instructions of the kit (Beyotime, Shanghai, China) [24]. The staining outcome was analyzed using the inverted microscope (Olympus, Tokyo, Japan).

### Alizarin red S staining

MC3T3-E1 was incubated with OM media containing 50 μM Trelagliptin for 14 days. The cells were then stained with 2% alizarin red dyes for approximately 10 minutes, followed by being washed with PBS buffer. The mineralized nodules were checked utilizing a phase-contrast microscopy [24,25].

### Statistical analysis

Results are shown as Mean ± standard deviation (SD). Differences were calculated with analysis of variance (ANOVA) followed by Bonferroni’s post-hoc test (SPSS 17.0 software). The difference was significant when P values < 0.05.

## Results

In the present study, using osteogenic medium (OM)-induced differentiated MC3T3-E1 cells, we examined the effect of the DPP-4 inhibitor Trelagliptin on differentiated Osteoblasts. We demonstrated that Trelagliptin enhanced the differentiation and mineralization by upregulating osteogenic gene expression via the AMPKα-RUNX2 axis.

### The effects of Trelagliptin on the cell viability of MC3T3-E1 cells

**Figure 1.**
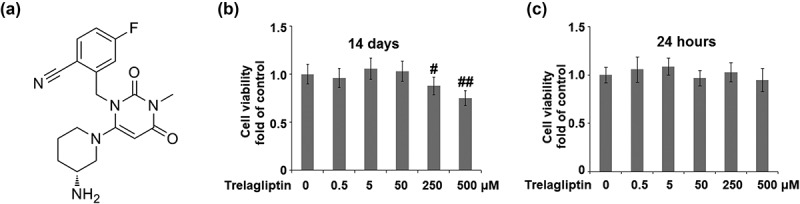
The effects of Trelagliptin on cell viability. (a). Molecular structure of Trelagliptin; (b-c). Cells were incubated with Trelagliptin (0, 0.5, 5, 50, 250, 500 μM) for 14 days or 24 hours. Cell viability was measured (#, ##, P < 0.05, 0.01 vs. vehicle group)

In a recent study by Liu et al, 25–100 μM Trelagliptin was used to treat mouse 3T3-L1 preadipocytes [26]. We tested the cytotoxicity of the osteoblastic cell line MC3T3-E1. To find the nontoxic dose, cells were incubated with Trelagliptin at the concentrations of 0, 0.5, 5, 50, 250, and 500 μM for 14 days, and the cell viability was determined using MTT assay on day 14. As shown in Figure 1(b), as the concentration of Trelagliptin increased from 0 to 50 μM, no significant difference was observed in the cell viability. However, when the concentration of Trelagliptin reached 250 μM, the cell viability decreased remarkably. At the higher concentrations of 250 and 500 μM Trelagliptin, treatment resulted in about 12% and 25% decrease in cell survival rate, respectively. As shown in Figure 1(c), these doses of Trelagliptin treatment for 24 hours did not show any influence on cell viability. Therefore, 50 μM was utilized as the safe concentration of Trelagliptin in the subsequent experiments to culture cells for 14 days.

### Trelagliptin promotes the differentiation of MC3T3-E1 cells

**Figure 2.**
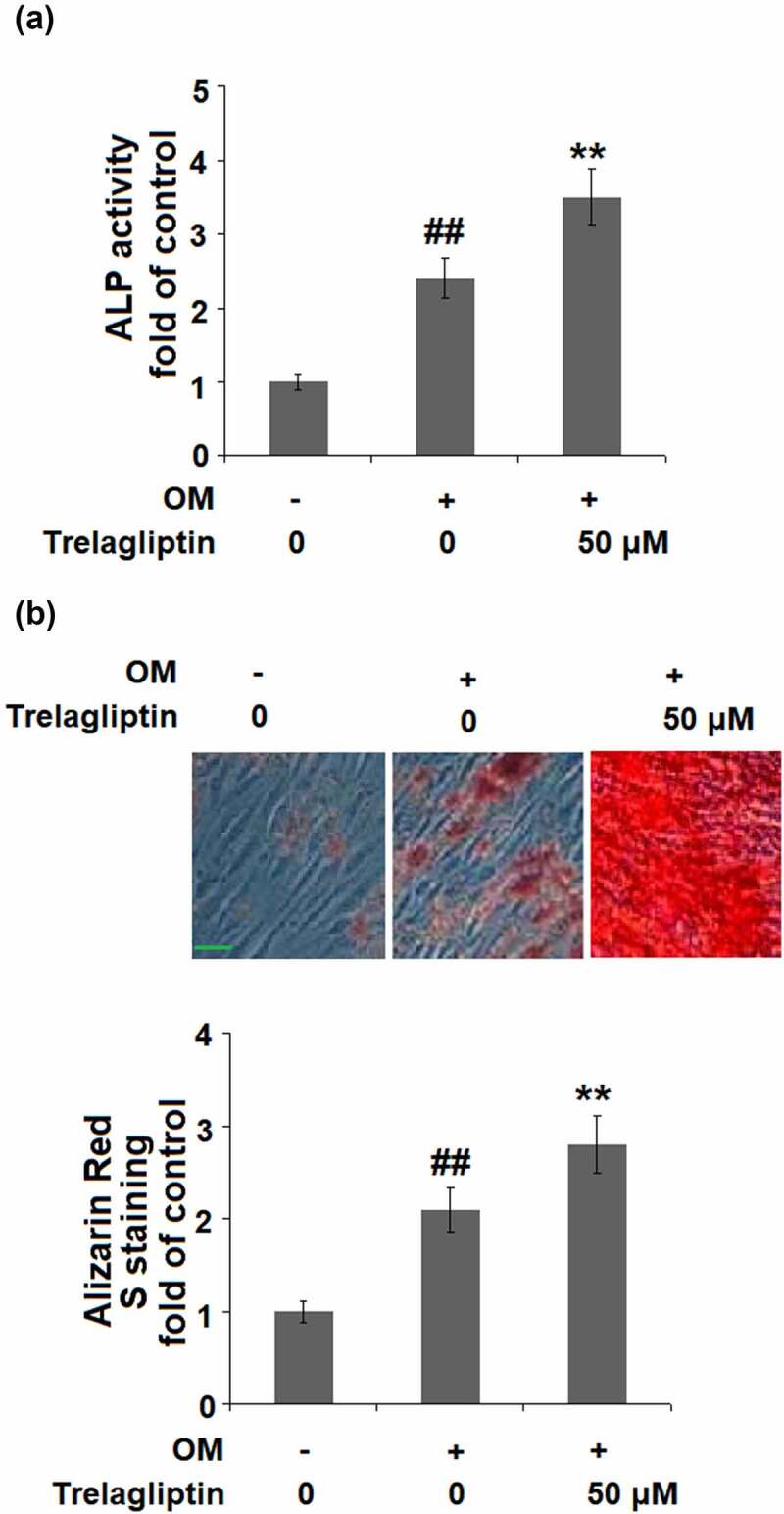
Trelagliptin promotes differentiation and mineralization. Cells were incubated with osteogenic medium (OM) with or without Trelagliptin (50 μM) for 14 days. (a). ALP activity; (b). Alizarin Red S staining. Scale bar, 100 μm (##, P < 0.01 vs. vehicle group; **, P < 0.01 vs. OM group)

The ALP activity was significantly elevated by the incubation with OM medium and further dramatically promoted by treatment with Trelagliptin (Figure 2(a)). In addition, the formation of calcified nodules (Figure 2(b)) was greatly increased in OM medium-incubated cells and further pronouncedly promoted by the introduction of Trelagliptin. These data indicate that Trelagliptin promoted the differentiation and mineralization of MC3T3-E1 cells.

### Trelagliptin induces the expression of marker genes of osteoblastic differentiation

**Figure 3.**
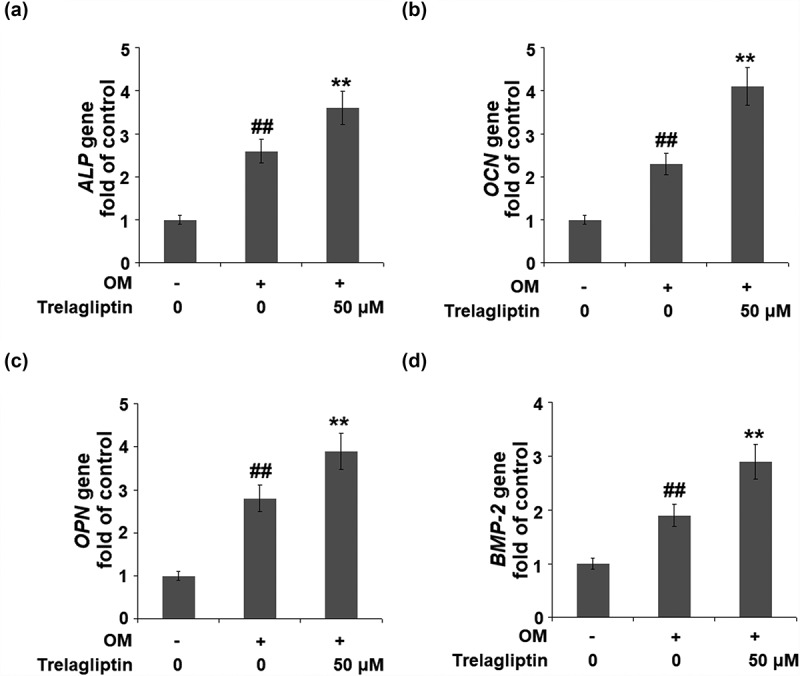
Trelagliptin increases the expression of marker genes of osteoblastic differentiation. Cells were incubated with OM with or without Trelagliptin (50 μM). (a). mRNA levels of *ALP*; (b). mRNA of *OCN*; (c). mRNA of *OPN*; (d). mRNA of *BMP-2* (##, P < 0.01 vs. vehicle group; **, P < 0.01 vs. OM group)

We further investigated the expression of osteoblastic differentiation biomarkers in the treated MC3T3-E1 cells. The gene expressions of *ALP, OCN, OPN*, and *BMP-2* in the MC3T3-E1 cells were significantly elevated by the incubation with OM medium and further upregulated by treatment with Trelagliptin (Figure 3(a–d)).

### Trelagliptin upregulates Runx2

**Figure 4.**
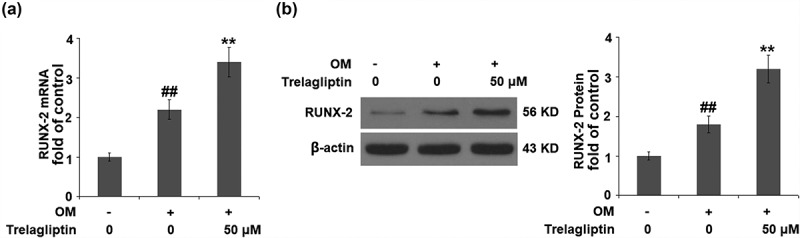
Trelagliptin increases the expression of RUNX2. Cells were incubated with OM with or without Trelagliptin (50 μM). (a). mRNA of RUNX2; (b). Protein of RUNX2 (##, P < 0.01 vs. vehicle group; **, P < 0.01 vs. OM group)

Runx2 is the vital transcriptional factor involved in the regulation of osteoblastic differentiation, the expression of which was evaluated. We found that the upregulated Runx2 induced by the incubation with OM was further upregulated by Trelagliptin (Figure 4), indicating a regulatory effect of Trelagliptin on Runx2.

### Trelagliptin promotes the phosphorylation of AMPKα

**Figure 5.**
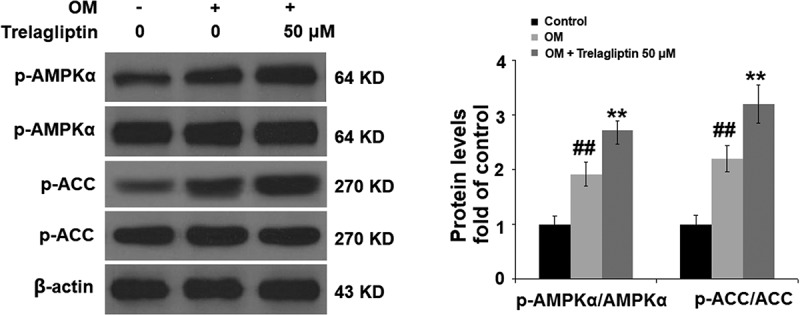
Trelagliptin increases the phosphorylation of AMPKα. Cells were incubated with OM with or without Trelagliptin (50 μM). The levels of p- AMPKα, AMPKα, p-ACC, ACC (##, P < 0.01 vs. vehicle group; **, P < 0.01 vs. OM group)

To further investigate the mechanism underlying the regulatory effect of Trelagliptin on Runx2, we examined the activity of the AMPK signaling pathway. As shown in Figure 5, no significant difference was observed on AMPKα and ACC when incubated with OM and Trelagliptin. Compared to the OM group, p-AMPKα and p-ACC were significantly elevated by treatment with Trelagliptin, indicating that the AMPK signaling pathway was significantly activated by Trelagliptin.

### Blockage of AMPK abolishes the effect of Trelagliptin

**Figure 6.**
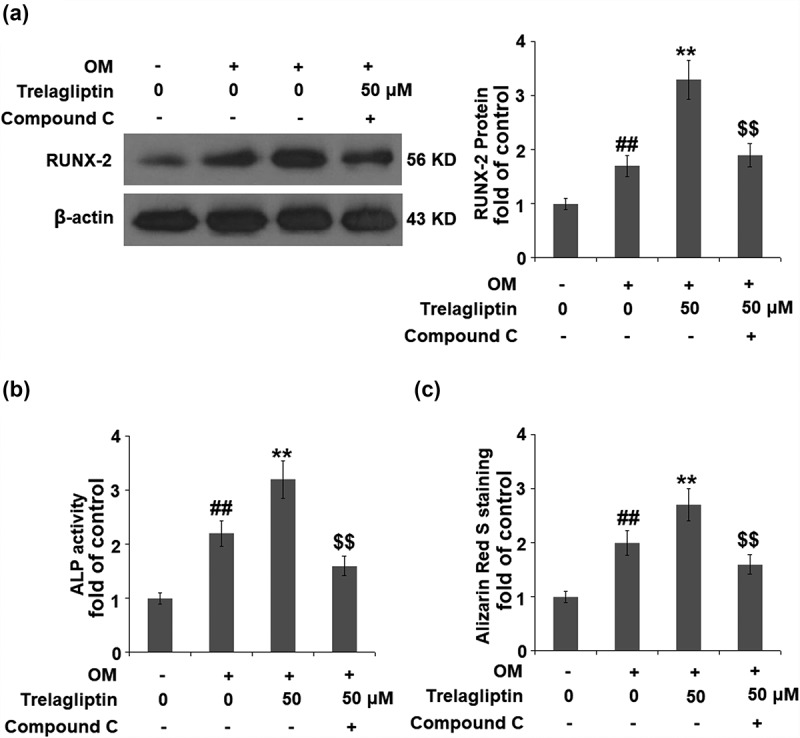
Inhibition of AMPK abolished the effect of Trelagliptin in the expression of Runx2 and MC3T3-E1 cell differentiation. Cells were incubated with OM with or without Trelagliptin (50 μM) or compound C for 14 days. (a). Protein level of RUNX2; (b). ALP activity; (c). Alizarin Red S staining (##, P < 0.01 vs. vehicle group; **, P < 0.01 vs. OM group; $$, P < 0.01 vs. OM+ Trelagliptin group)

To confirm that the biological effect of Trelagliptin is related to AMPK, cells were stimulated with OM with or without Trelagliptin (50 μM) or compound C, which is an inhibitor of the AMPK signaling pathway. The elevated expression of Runx2 induced by Trelagliptin was significantly suppressed by the co-treatment with compound C (Figure 6(a)). Also, the activated ALP activity (Figure 6(b)) and increased formation of calcified nodules (Figure 6(c)) induced by Trelagliptin were both dramatically reversed by the co-treatment of compound C. These data indicate that the effects of Trelagliptin on the expression of Runx2 and the differentiation of MC3T3-E1 cells were mediated by the activation of the AMPK signaling pathway.

## Discussion

Runx2 consists of 1 Runt structural domain, 1 transcriptional repression zone, 1 nuclear localization signal region, and 3 transcriptional activation zones. The Runt structural domain initiates the expression of osteoblasts-related genes by binding to the osteoblasts’ cis-acting elements [27,28]. The differentiation and proliferation of osteoblasts can be regulated by multiple transcriptional factors, such as leucine zipper protein, and zinc finger protein. Runx2 not only plays an important role in the differential processing of BMSCs to osteoblasts but also promotes the maturation procedure of osteoblasts [29], Otto [30] and Komori [31] reported that Runx2^−/-^ mice were frail after birth. The results of ALP staining and X-ray assay show that calcified tissue in the femurs of Runx2^−/-^ mice was completely absent. In addition, in the skulls and femurs of Runx2^−/-^ mice, Col1α1, an important biomarker in the early stage of osteoblasts, was found to be unexpressed, indicating an indispensable role of Runx2 in the intramembranous ossification and endochondral ossification. Takarada performed Alizarin red and Alcian blue staining on the skeletons of Runx2^−/-^ mice. The blue staining, which represented cartilage, was observed, indicating that the skeletons mainly consisted of cartilage. However, the red staining, which represented the calcified tissues, was not observed [32]. Runx2 is also regarded as an important target gene for the regulation of the osteogenesis differentiation of BMSCs. Byers reported that the ability of osteogenesis differentiation was found to be elevated significantly after upregulating the expression of Runx2 in the rat BMSCs. More Col1 and calcium nodules were observed after transferring the Runx2-overexpressed BMSCs onto the three-dimensional scaffolds [33]. In the present study, we firstly found that the differentiation of MC3T3-E1 cells was dramatically promoted by treatment with Trelagliptin and verified by activated ALP and increased calcium nodules. Also, the expressions of the biomarkers of osteoblastic differentiation, ALP, OCN, OPN, and BMP-2 [34,35], were found to be greatly upregulated by treatment with Trelagliptin, indicating the inductive effect of Trelagliptin on osteoblastic differentiation. However, our data indicate that Trelagliptin alone did not have any influence on the expressions of osteoblast differentiation markers *ALP, OCN, OPN, and BMP-2* when the cells were grown in basal growth media (Supplementary Figure 1), suggesting soluble osteogenic factors in osteogenic media are required for the modulation of Trelagliptin on osteogenic differentiation. At the same time, Runx2 was also found to be significantly upregulated in the MC3T3-E1 cells by the introduction of Trelagliptin, indicating that Trelagliptin might exert a promising positive effect against differential processing from BMSCs to osteoblasts and maturation procedure of osteoblasts. However, more evidence will be provided by our future work to verify the therapeutic effect of Trelagliptin against osteoporosis, including evaluating the property of Trelagliptin in a postmenopausal osteoporosis animal model.

AMPK is composed of α, β, and γ subunits, and its activation initiates the catabolic pathway and closes the anabolic pathway [36,37]. Kanazawa [38] reported that 5-Aminoimidazole-4-carboxamide ribonucleotide (AICAR) induced differentiation in the MC3T3-E1 cells by activating AMPK. The mineralization in the autocrine or paracrine pathway within osteoblasts can also be induced by type I adiponectin receptor and the AMPK signaling pathway. Lee found that the differentiation of osteoblasts induced by BMP could be blocked by Compound C, accompanied by the downregulation of osteogenic marker genes, including *Runx2, Osx*, and *Ocn* [39]. Acetyl-coA carboxylase (ACC) is an important down-stream enzyme of AMPK involved in the regulation of bone metabolism [40]. Here, we report that the AMPK/ACC signaling pathway was significantly activated in MC3T3-E1 cells by the introduction of Trelagliptin, indicating a regulatory effect of Trelagliptin on the AMPK/ACC signaling pathway-mediated bone metabolism. We further introduced compound C, an AMPK inhibitor, into the experimental system and found that the effects of Trelagliptin in the expression of Runx2 and the differentiation of MC3T3-E1 cells were all blocked by compound C, indicating that Trelagliptin induced the osteoblast differentiation by mediating the AMPK/ACC signaling pathway. In our future work, the specific target of Trelagliptin will be investigated to better understand the regulatory effect of Trelagliptin on the AMPK/ACC signaling pathway, as well as the therapeutic property of Trelagliptin on osteoporosis.

## Conclusion

Taken together, our data show that the long-lasting DPP-4 inhibitor Trelagliptin stimulates osteoblastic differentiation by upregulating transcriptional regulator Runx2 through activating the AMPK/ACC signaling pathway. This evidence implies that Trelagliptin possesses the potential effect to modulate bone metabolism, and it could have therapeutic potential in the modulation of diabetes related-osteoporosis.

## Supplementary Material

Supplemental Material
