# Supplementary material for: Trelagliptin stimulates osteoblastic differentiation by increasing runt-related transcription factor 2 (RUNX2): a therapeutic implication in osteoporosis
Source: Bioengineered. 2021 Mar 18;12(1):960–8. doi: 10.1080/21655979.2021.1900633 (PMC8291811; doi:10.1080/21655979.2021.1900633)
Supplement: Supplemental Material [file KBIE_A_1900633_SM2168.zip › Document.rtf]

Supplementary Figure 1. The effects of Trelagliptin alone on the gene expression of marker genes of osteoblastic differentiation. Cells were incubated with Trelagliptin (50 ìM) alone in basal growth media. (A). mRNA levels of ALP; (B). mRNA of OCN; (C). mRNA of OPN; (D). mRNA of BMP-2.
