# Supplementary figures and images for: Trelagliptin stimulates osteoblastic differentiation by increasing runt-related transcription factor 2 (RUNX2): a therapeutic implication in osteoporosis
Source: Bioengineered. 2021 Mar 18;12(1):960–8. doi: 10.1080/21655979.2021.1900633 (PMC8291811; doi:10.1080/21655979.2021.1900633)

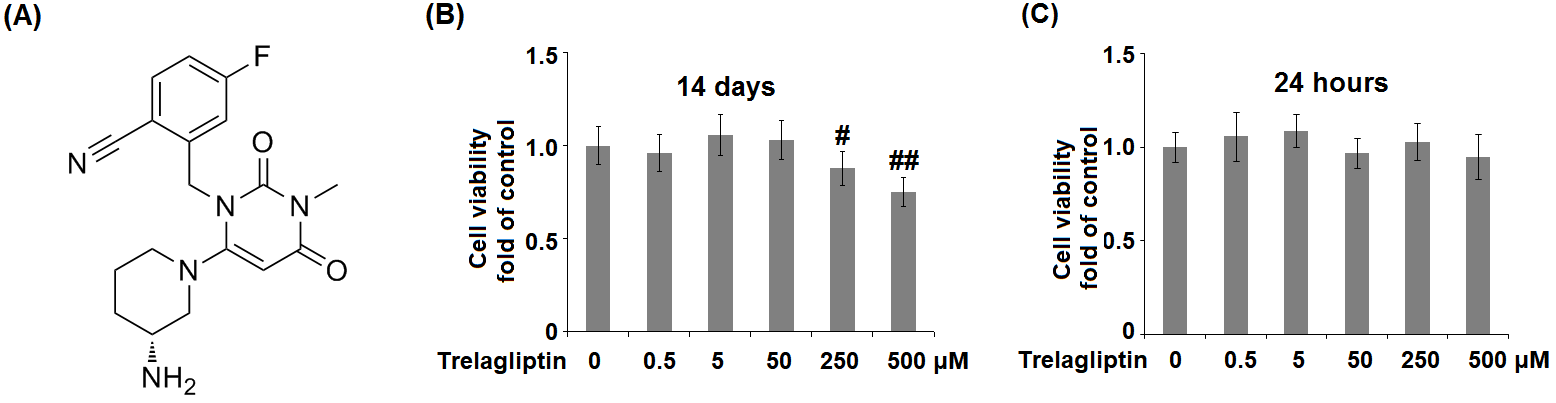

Supplement: Supplemental Material [file KBIE_A_1900633_SM2168.zip › Supplementary Fig 1.tif]
